# A genetic replacement system for selection-based engineering of essential proteins

**DOI:** 10.1186/1475-2859-11-110

**Published:** 2012-08-16

**Authors:** Sonja Billerbeck, Sven Panke

**Affiliations:** 1 ETH Zürich, Department for Biosystems Science and Engineering (D-BSSE), Mattenstrasse 26, 4058, Basel, Switzerland

## Abstract

**Background:**

Essential genes represent the core of biological functions required for viability. Molecular understanding of essentiality as well as design of synthetic cellular systems includes the engineering of essential proteins. An impediment to this effort is the lack of growth-based selection systems suitable for directed evolution approaches.

**Results:**

We established a simple strategy for genetic replacement of an essential gene by a (library of) variant(s) during a transformation.

The system was validated using three different essential genes and plasmid combinations and it reproducibly shows transformation efficiencies on the order of 10^7^ transformants per microgram of DNA without any identifiable false positives. This allowed for reliable recovery of functional variants out of at least a 10^5^-fold excess of non-functional variants. This outperformed selection in conventional bleach-out strains by at least two orders of magnitude, where recombination between functional and non-functional variants interfered with reliable recovery even in *recA* negative strains.

**Conclusions:**

We propose that this selection system is extremely suitable for evaluating large libraries of engineered essential proteins resulting in the reliable isolation of functional variants in a clean strain background which can readily be used for *in vivo* applications as well as expression and purification for use in *in vitro* studies.

## Background

About eight percent of *E. coli* genes are essential for the cell [1]. Essential genes are of particular scientific interest as they encode proteins required for important biological functions, thereby building the minimal core of cellular viability which tends to be conserved across species. Knowledge about essential genes and their protein products is important for drug design [2,3], biotechnological applications [4], minimal genome approaches [5-8] and, in general, crucial for understanding and engineering the basic cellular functions required for life [9]. While the construction of the Keio-collection, a collection of single gene knock-outs in *E. coli*[1], enormously facilitated the systematic investigation of the physiology of *E. coli* as well as protein and strain-engineering approaches, it is still restricted to non-essential genes and their protein products. Engineering approaches involving essential genes and proteins are complicated because knock-outs cause lethality. This means that phenotypes of engineered proteins cannot be easily evaluated *in vivo* as suitable clean strain backgrounds are not available.

In the last decade several approaches have been investigated to identify essential genes and to study their function *in vivo* by conditional elimination of the protein from the cell. This was achieved by triggering interference of the synthesis of the target protein on either the transcriptional or translational level [10-15]. However, these “bleach-out” methods rely on conditional protein elimination rather than elimination of the target gene itself and thus retain a wild-type copy of the essential gene in the cell. This sets limitations for the utility of these systems as ready-to-use selection systems for directed evolution experiments since recombination of library members with the chromosomal wild-type gene or mutations in the system regulating the expression of the wild-type protein can lead to the selection of false positive variants. This is particularly true when using a library for which only a small fraction of variants is expected to be functional. In this case recombination events are preferentially selected over functional library members, which results in every selection effort turning into a laborious screen for bona fide functional library members.

Besides evaluation of large libraries, another desire during protein engineering of essential genes is to replace the wild-type gene by a single engineered or heterologous variant for *in vivo* functional studies or for the construction of specialized strains which can be used to purify the mutant protein free of wild-type protein. Phage P1-mediated transduction of a chromosomal knock-out into a strain expressing a variant of the essential gene of interest from a plasmid is the current method of choice to achieve genetic replacement of an essential target gene by a variant (e.g [16]). The knock-out was thereby created while complementing the chromosomal gene loss by a plasmid-encoded version of the essential gene.

Although P1-transduction is widely used, the protocol is time-consuming and restricted to a few variants at a time as efficiencies of successful transductions are low, often requiring empirical testing for the proper phage concentration followed by re-plating and PCR-screening for correct genotypes. Therefore, it was our aim to develop a general genetic set-up which turns working with essential genes and the engineering of their gene products into a straight-forward approach as facile as working with non-essential genes. Here, we present a simple transformation-based system. Establishment of the system begins with the chromosomal replacement of the essential gene of interest by a PCR-derived selection marker [17], in conjunction with a complementary vector-encoded version of the target. A central element of the method is that the complementation vector carries an I-*Sce*I nuclease recognition site and can thus be rapidly and conditionally eliminated in the presence of an I-*Sce*I nuclease-expressing helper plasmid. During elimination of the complementation vector, cells are made electrocompetent and transformed with a vector-encoded library or a specific variant of the essential gene. Thus, the actual step of gene exchange is reduced to a single transformation plus preparation of a suitable knock-out strain.

## Results

### Overview of the replacement system

**Figure 1:**
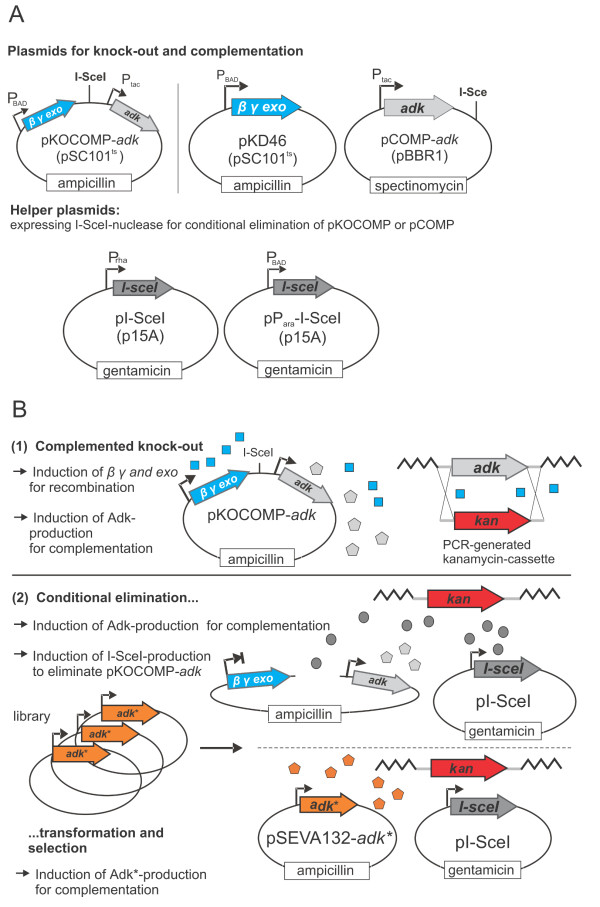
**Overview on the transformation-based replacement of essential genes. A**: Plasmids required for establishment of the selection system **B**: General procedure: The essential target gene *adk* is replaced by an antibiotic resistance cassette while pKOCOMP-*adk* complements for the chromosomal loss. Plasmid pKOCOMP can be conditionally eliminated by co-expression of I-*Sce*I nuclease from helper plasmid pI-*Sce*I. During elimination, cells are made electrocompetent and are transformed with a variant or library under investigation to evaluate functionality or to select for functional library members which can complement for the loss of pKOCOMP.

The selection system can be flexibly assembled in various user-defined ways, but will be discussed in its simplest version first. It relies on two vectors: the knock-out and complementation vector pKOCOMP and the helper plasmid pI-*Sce*I (Figure 1A and Table 1). Plasmid pKOCOMP is a derivative of pKD46 [17] and encodes the arabinose-inducible λ *red* recombination system (genes *βγ* and *exo*) as well as the essential gene of interest. It also contains an 18 bp I-*Sce*I cleavage site which allows for the conditional elimination of pKOCOMP in the presence of I-*Sce*I. I-*Sce*I is expressed from the helper plasmid pI-*Sce*I under the control of the rhamnose-inducible RhaSR/P_*rha*BAD_-system [18]. A variant of this helper plasmid, pP_*ara*_I-*Sce*I, carries an arabinose-inducible regulatory system AraC/P_*ara*BAD_[19] instead of the rhamnose-based system. After testing various set-ups, these promoters were chosen as they exhibited high expression level in the presence of rhamnose or arabinose but could be efficiently switched off in the presence of glucose due to catabolite repression [19,20]. It should be noted that the specific set-up of the complementation vector is flexible as long as it carries the I-*Sce*I site, which can easily be introduced by PCR, is compatible with pKD46, which is required for λ red-mediated knock-out of the target gene and can be cured by growth at 42°C. The system can then be completed by subsequent transformation with pI-*Sce*I or pP_*ara*_I-*Sce*I. 

**Table 1:** Bacterial strains and plasmids used in this study

**Bacterial strain or plasmid**	**Characteristics**	**Source**
**Strains**		
W3110	F^-^ λ^-^*rph-1* INV(*rrnD*, *rrnE*)	[33], internal strain collection
DH10B	F^-^*endA1 recA1 galE15 galK16 nupG rpsL ΔlacX74 Φ80lacZΔM15 araD139 Δ(ara,leu)7697 mcrA Δ(mrr-hsdRMS-mcrBC)* λ^-^	[34], internal strain collection
W3110 *adk::kan*	Chromosomal *adk* replaced by a Km^R^ cassette. The strain is only viable if the *adk* deletion is complemented.	This work
W3110 *groE::kan*	Chromosomal *groS* and *groL* replaced by a Km^R^ cassette. The strain is only viable if the *gro*E deletion is complemented.	This work
W3110 *secB-gpsA::kan*	Chromosomal *secB and gpsA* replaced by a Km^R^ cassette. The strain is only viable if the *gps*A deletion is complemented.	This work
SBΔrecA	*lacI*^*q*^*rrnB3* Δ*lacZ4787 hsdR514* Δ (*araBAD*) *567* Δ (*rhaBAD*) *568 rph-1 groE*::P_*araBAD*_*-groE-Km*^*R*^*recA::FRT*	This work
**Plasmids**		
pKD46	*ori* pSC101^ts^, Ap^R^. Encodes λ red recombination genes *γ, β and exo* under control of the arabinose-responsive promoter P_*ara*BAD_.	[1]
pKOCOMP-adk	pKOCOMP-derived vector with *adk* under control of an IPTG-responsive *tac* promoter	This work
pSEVA432	*ori* pBBR1, Spec^R^, multiple cloning site	provided by Victor de Lorenzo
pCOMP-adk	pSEVA432 encoding for *adk* under control of an IPTG-responsive *tac* promoter, contains an I-*Sce*I cleavage site	This work
pCOMP-ESL	pSEVA432 encoding for *groS* and *groL* control of an IPTG-responsive *tac* promoter, contains an I-*Sce*I cleavage site	This work
pCOMP-*sec*B*-gps*A	pSEVA432 encoding for the natural *secB-gpsA* transcriptional unit, contains a I-*Sce*I cleavage site	This work
pSEVA132	*ori* pBBR1, Ap^R^, multiple cloning site	provided by Victor de Lorenzo
pSEVA132-*adk*	pSEVA132 encoding for *adk* under control of its natural promoter	This work
pSEVA132-*adk*^Stop^	pSEVA132-*adk* with an internal stop codon	This work
pSEVA132-*adk*^watermark^	pSEVA132-*adk* with a peptide insertion behind position P140	This work
pSEVA132-*gro*E	pSEVA132 coding for *groS and groL* under control of their natural promoter	This work
pSEVA132-*gro*E^Stop^	pSEVA132-*groE* with an internal stop codon	This work
pSEVA132-*gro*E^watermark^	pSEVA132-*groE* with a peptide insertion behind site I301	This work
pSEVA132-*sec*B*gps*A	pSEVA132 encoding for *secB and gpsA* under control of their natural promoter	This work
pSEVA132- *sec*B*gps*A^Stop^	pSEVA132- *sec*B*gps*A with an internal stop codon	This work
pSEVA671	ori p15A, Gm^R^, multiple cloning site	provided by Victor de Lorenzo
pI-*Sce*I	pSEVA671, with I-*Sce*I nuclease under control of the rhamnose inducible promoter P_Rha_ and the response regulators RhaS and RhaR, derived from the rhammnose metabolizing transcriptional unit of *E. coli.*	This work
pP_ara_I-*Sce*I	pI-*Sce*I with P_rhaBAD_ and regulators RhaS and RhaR exchanged by the arabinose promoter P_*ara*BAD_ and the regulator *araC*	This work

### Validation and characterization of the system using the essential gene product adenylate kinase (Adk)

**Figure 2:**
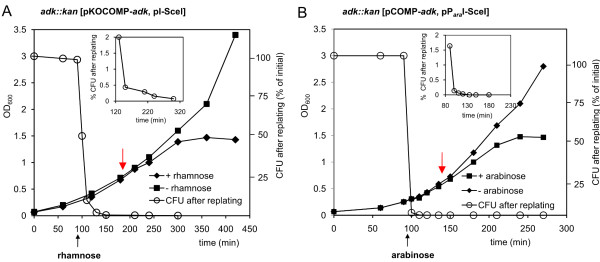
**Characterization of the pKOCOMP-*adk*/pI-*Sce*I and the pCOMP-*adk*/pP**_***ara***_**I-*Sce*I systems. A**: Elimination of pKOCOMP-*adk from adk::kan* due to rhamnose-induced expression of I-*Sce*I nuclease from helper plasmid pI-*Sce*I. Red arrow: Time point for harvest and competent cell preparation. The inlet gives a better resolution of the elimination dynamics. The chromosomal *adk*-replacement was established using pKOCOMP-*adk.***B**: Elimination of pCOMP-*adk* from *adk::kan* due to arabinose-induced expression of I-*Sce*I nuclease from helper plasmid pP_*ara*_I-*Sce*I. The chromosomal *adk*-replacement was established using pKD46 and pCOMP-*adk.*

The first target gene chosen for validation and characterization of the system was *adk,* encoding *E. coli’*s Adk, an essential gene product required for the biosynthesis of purine ribonucleotides and for the regulation of intracellular nucleotide availability [1,21,22]*.* For complementation, *adk* under control of P_*tac*_ was inserted into pKOCOMP, giving rise to pKOCOMP-*adk*. Upon induction of pKOCOMP-*adk* with IPTG, the chromosomal copy of *adk* was replaced by a PCR-generated kanamycin resistance cassette [17]. The genotype of the resulting strain *E. coli adk::kan* [pKOCOMP-*adk* was confirmed by PCR using primers binding to chromosomal regions up- and downstream of the *adk*-locus ( Additional file 1: Table S1 and Additional file 2 Figure S1). To complete the selection system, strain *adk::kan* [pKOCOMP-*adk* was transformed with helper plasmid pI-*Sce*I. The resulting strain *adk::kan* [pKOCOMP-*adk*; pI-*Sce*I] was grown in the presence of glucose during maintenance to repress I-*Sce*I nuclease production. However, when cells were transferred to glucose-free LB medium, I-*Sce*I production was apparently efficiently induced by the addition of rhamnose, as no colony-forming units could be recovered after an induction-8period of 180 minutes (Figure 2A). Importantly, even without a copy of the *adk* gene, cells remained viable for about two generations, presumably until all mRNA and protein was depleted. This was expected as linear DNA is rapidly degraded by cellular exonucleases and has a half-life in the range of minutes [23] whereas most proteins are relatively stable with half-lives in the range of hours [24]. 

During this intermediate period cells were made electrocompetent by washing with H_2_O and glycerol and transformed with various test plasmids (Table 1) in order to determine transformation efficiencies and the frequency of false positive variants. Vector pSEVA132-*adk* encoding the wild-type adenylate kinase under control of its natural promoter was used for determination of transformation efficiencies. Transformation efficiencies of 10^7^ μg^-1^ DNA were routinely achieved. The system was intensively characterized regarding the frequency of false positive variants which could arise due to recombination of the library plasmid with residual linearized pKOCOMP-*adk* or due to an uninduced subpopulation of cells that maintained pKOCOMP (Table 2). Recombination-based false positives were tested by transformation with pSEVA132-*adk*^*stop*^, harboring a stop codon in the *adk* gene that could be repaired by recombination allowing growth of the corresponding carrier cell. False positives due to incomplete induction were examined by transformation with the empty vector pSEVA132. In both cases no false positive colonies were detected when I-*Sce*I expression was induced and pKOCOMP was eliminated before competent cell preparation (Table 2), corresponding to a frequency of less than 2 x 10^-4^ false positives per transformed cell. However, there was a detectable frequency of recombination events when pKOCOMP was eliminated only after transformation with the test plasmids or when we tried to eliminate pKOCOMP solely by growth at the non-permissive temperature. In the former case, I-*Sce*I nuclease expression was induced only after transformation by plating on arabinose-supplemented agar plates, such that both plasmids would be simultaneously present in the cells for a short period of time. Here we found illegitimate events with a frequency of about 3 x 10^-3^ per transformed cell (Table 2). This demonstrates the importance of careful management of the plasmid elimination step.

**Table 2:** Transformation efficiencies and frequency of false positive variants for the Adk-specific selection systems

**Strain**	**I-*Sce*I induction during competent cell preparation**	**Number of transformants (frequency CFU per transformant)**		
		**pSEVA132-adk**	**pSEVA132-adk**^**stop**^	**pSEVA132**
**W3110*adk::kan*[pKOCOMP-*adk*, pI-*Sce*I]**	No	20 200 ± 2900	66 ± 21 (~3x10^-3^)	28 ± 13 (~1x10^-3^)
	Yes	21 000 ± 6000	0 (<2x10^-4^)	0 (<2 x10^-4^)
**W3110*adk*::kan [pCOMP-*adk,*pP**_***ara***_**I-*Sce*I]**	No	32 100 ± 3700	61 ± 17 (2x10^-3^)	35 ± 11 (1x10^-3^)
	Yes	28 000 ± 2200	0 (<2x10^-4^)	0 (<2x10^-4^)

### Flexibility of the replacement system

To verify that the selection system can be set up with alternative combinations, we constructed pCOMP-*adk*. This vector is based on the pBBR1 *ori* with an expected copy number of 10-20 per cell [25], carrying a P_*tac*_ promoter-controlled *adk* gene, the *lac*-repressor LacI, and an I-*Sce*I cleavage site. While the previous complementation vector pKOCOMP-*adk* used a temperature-sensitive pSC101 *ori* with about 2-3 copies per cell when grown at 37°C [26], the increased copy number of pCOMP-*adk* allowed for examination of whether plasmid elimination was sufficiently efficient at higher intracellular plasmid concentration. We used pCOMP-*adk* in combination with pKD46 to replace *adk* by a kanamycin cassette. After curing cells of pKD46 by growth at 43°C, the resulting strain *adk::kan* [pCOMP-*adk* was transformed with pP_*ara*_I-*Sce*I and used for gene replacement as described before using arabinose to induce I-SceI production. Despite the higher copy number of pCOMP, the plasmid was again rapidly eliminated from the cells in the presence of arabinose (Figure 2B). Probably due to the faster on-set and the possibly higher expression levels of the arabinose responsive promoter P_*ara*BAD_[19,20], elimination-dynamics of the pCOMP/pP_*ara*_I-*Sce*I system were faster than those of the pKOCOMP/pI-*Sce*I system. Although the elimination data of the two systems are difficult to compare due to differences in copy number and promoters, these results indicate that the described approach can be set up in multiple ways, making it easy to adapt to plasmid strategies for specific purposes. Transformation efficiencies of the pCOMP/pP_*ara*_I-*Sce*I system (~10^7^ colonies μg^-1^ DNA) were comparable to the pKOCOMP/pI-*Sce*I system (Table 1) and we could not identify false positive transformants.

### Generality of the system

To confirm that the utility of the system was not limited to *adk* but could be easily extended to other essential genes, we constructed *in vivo* selection systems for other essential gene products: the chaperonin GroEL and its co-chaperonin GroES (encoded by the *groE* operon containing the genes *groL* and *groS*), and glycerol-3-phosphate dehydrogenase (GpsA encoded by *gpsA*). For establishment of the GroEL-specific system we introduced an I-*Sce*I cleavage site into the vector pSEVA431-*groE* by PCR giving rise to vector pCOMP-*groE*. Plasmid pSEVA431-*groE* encodes the *groE*-operon under control of the IPTG-inducible P_*tac*_ promoter. It also harbors the *lacI* gene, a spectinomycin resistance cassette and replicates with a pBBR1 *ori*. Plasmid pCOMP-*groE* was used in combination with pKD46 to replace the chromosomal *groE*-operon by a kanamycin resistance cassette ( Additional file 2 Figure S1). The resulting strain *groE::kan* [pCOMP-*groE*] was cured from pKD46 at 43°C and then transformed with the helper plasmid pP_*ara*_I-*Sce*I.

**Figure 3:**
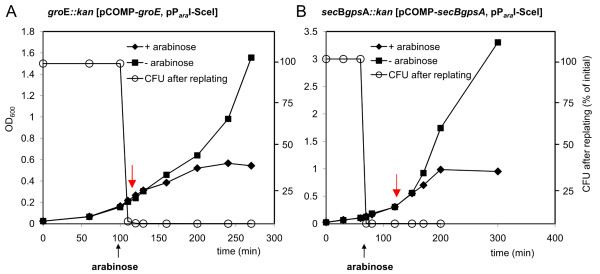
**Characterization of the pCOMP-*groE*/pP**_***ara***_**I-*Sce*I and the pCOMP-sec*BgpsA*/pP**_***ara***_**I-*Sce*I system.** Elimination of (**A**) pCOMP-*groE* from *groE::kan* and (**B**) pCOMP-*secBgpsA* from *secBgpsA::kan* in the presence of arabinose and the helper plasmid pP_ara_I-*Sce*I. Elimination is induced by arabinose-induced expression of I-*Sce*I nuclease from helper plasmid pP_ara_I-*Sce*I. Red arrow: Time point for harvest and competent cell preparation.

The GpsA-specific system was constructed by cloning the natural *gpsA* transcriptional unit (consisting of genes *secB* and *gpsA* under control of their natural promoter) into vector pSEVA431 using primers encoding for an I-*Sce*I restriction site, resulting in vector pCOMP-*secBgpsA*. Chromosomally encoded *secB* and *gpsA* were then replaced by a kanamycin resistance cassette using pKD46 ( Additional file 2 Figure S1). To complete the set-up, strain *secBgpsA::kan* [pCOMP-*secBgpsA*] was cured of pKD46 and transformed with helper plasmid pP_*ara*_I-*Sce*I. Both systems were characterized regarding elimination dynamics of the complementing plasmids pCOMP-*groE* and pCOMP-*secBgpsA* after I-*Sce*I induction, as well as regarding transformation efficiencies of electrocompetent cells prepared during pCOMP-elimination and frequency of false positive variants. Both pCOMP-type plasmids were lost at a comparable rate to the pCOMP-plasmid carrying *adk* (Figure 3). After that, both systems routinely showed transformation efficiencies of 10^6^-10^7^ colonies μg^-1^ DNA when transformed with the positive control vectors pSEVA132-*groE* or pSEVA132-*secBgpsA*. No false positive variants could be detected after transformation with the test plasmids pSEVA132-*groE*^*stop*^ and pSEVA132-*secBgpsA*^*stop*^, constructed in analogy to pSEVA132-*adk*^*stop*^ before. Importantly, it seems to be a general feature that cells stay viable - as judged by the doubling time in comparison to a non-induced control culture that did not produce I-*Sce*I - for one or more generations, depending on the target gene, after loss of the complementing plasmid. This is an important characteristic of the system as cells can be made competent for transformation with a library or variant of an essential gene during a period where the complementing plasmid has already been lost and can no longer contribute to recombination.

### Performance of the system during selection

To test the system regarding its performance in reliably identifying functional variants from a large library without contamination by false positive variants we challenged the GroEL- and the Adk-specific selection system with mock libraries with various excesses of non-functional variants. As there is a conventional bleach-out system available for GroEL (the *E. coli* MGM100 strain [10]), we also compared the performance of the replacement system developed here to the bleach-out system. In strain MGM100*,* the *groE* promoter has been replaced by the *araBAD* promoter and the regulatory gene *araC*. The strain can therefore be maintained in the presence of arabinose but GroEL production from the chromosomal locus can be fully repressed in the presence of glucose. This way, functional variants can be selected from a library in the presence of glucose. However, as a copy of the wild-type gene is still present during selection, recombination can lead to selection of false positive variants, which can dominate even in stringent selection conditions. To improve the strain regarding the expected recombination frequency we deleted *recA* leading to the strain SBΔ*recA* ( Additional file 2: Figure S1).

The created mock libraries contained decreasing ratios of functional to non-functional gene variants from 1:10^2^ to 1:10^5^ (functional:non-functional) mimicking libraries where a decreasing number of variants is expected to be functional within a large pool of non-functional variants, as it is typical e.g. for libraries created by error-prone PCR with different error rates. As non-functional variants we used pSEVA132-*groE*^*stop*^ or pSEVA132-*adk*^*stop*^. As functional variants we used pSEVA132-*groE*^*watermark*^ or pSEVA132-*adk*^*watermark*^. These plasmids encode for a GroEL or Adk*-*variant with an in-frame insertion of a short peptide at previously identified permissive sites. Both variants are fully functional and can be identified by PCR using watermark-specific primers. To compare the GroEL-specific selection system with the GroEL bleach-out system SBΔ*recA*, electrocompetent W3110 *groE::kan* [pCOMP-*groE*, pP_*ara*_I-*Sce*I] cells or electrocompetent SBΔ*recA* cells were transformed with the different mock libraries in separate experiments. As positive and negative controls, cells were transformed with only functional or only non-functional variants. After plating and incubation overnight on LB-agar plates supplemented with arabinose, the corresponding antibiotics, and, in the case of W3110 *groE::kan* [pCOMP-*groE,* pP_*ara*_I-*Sce*I], glucose, colonies were scored and a subset of the colonies was genotyped with watermark-specific primers (Table 3).

**Table 3:** Recovery of functional GroEL-variants and Adk-variants from mock libraries with increasing excess of non-functional variants using the established selection system in comparison with a conventional bleach-out system

**Strain**	**Library (functional: non-functional)**	**Number of colonies after transformation**^**a**^	**Number of genotyped variants**	**Number of detected false positive variants**
W3110 *groE*::kan [pCOMP-*groE*, pP_*ara*_I –*Sce*I]	Only functional	~ 27 000	20	0
	1:10^2^	231	20	0
	1:10^3^	33	20	0
	1:10^4^	4	4	0
	1:10^5^	1^a^	1	0
	Only non-functional	0	0	0
SBΔ*recA*	Only functional	~ 25 000	20	0
	1:10^2^	253	20	0
	1:10^3^	25	20	1
	1:10^4^	7	7	5
	1:10^5^	6	6	5
	Only non-functional	7	7	7
W3110 *adk*::kan [pCOMP-*adk,* pP_*ara*_I-*Sce*I]	Only functional	~ 46 000	20	0
	1:10^2^	459	20	0
	1:10^3^	36	20	0
	1:10^4^	3	3	0
	1:10^5^	1	1	0
	Only non-functional	0	0	0

^a^Typically, 10^9^ cells were used in the transformation, which would in some cases not have allowed to isolate a positive colony in the high-stringency case. In these cases, the number of transformed cells was increased to 3*10^9^ cells.

In the GroEL-specific system, the numbers of colonies correlated well with the numbers expected from transformation efficiencies and the decreasing number of functional variants in each library. Even from the most stringent library (functional variants at a frequency of 10^-5^) only functional variants carrying the watermark were recovered after transformation of ~ 81,000 cells. No colony was detected after transformation of approximately the same number of cells with only non-functional variants. In contrast when using SBΔ*recA* for the same experiment, false positive variants were detected after transformation with the negative control (transformation with only non-functional variants) with a frequency of ~ 2 x 10^-4^. In addition, we identified false positive variants after genotyping a subset of the colonies which had been selected from the different mock libraries (Table 3). Selection stringency positively correlated with the false positive rate, thus requiring laborious orthogonal assays to differentiate true from false positives. In a directed evolution experiment this would result in the requirement for intensive post-screening of selected variants for true functional library members. Plasmids of three of the false positive variants, which had been isolated after transformation with only non-functional variants, were further analyzed by sequencing. For two of them the stop codon had been reverted to the wild-type codon, probably due to recombination with the chromosomal *groL* copy. The third analyzed variant still had the stop codon within *groL* indicating that the ability to grow must have arisen from a mutation in the *araBAD* promoter preventing full repression. This phenomenon had been described before for strain MGM100 [16].

The same experiment was performed with the Adk-specific selection system using mock libraries with decreasing ratios of functional Adk-variants to non-functional Adk-variants. Also for this set-up we did not identify any false positive variant and after transformation with the most stringent library we could reliably identify a functional variant containing the watermark after transformation of ~ 138,000 cells (Table 3).

## Discussion

In this study we present a facile and efficient set-up for a (within the tested boundaries) background-free selection system for functional, engineered, essential proteins. It is based on the conditional elimination of a complementary plasmid-based copy of an essential gene in a knock-out strain in order to replace the essential gene by genes from a library (for example).

We show that I-*Sce*I nuclease-based cleavage proves to be a suitable strategy for the fast and efficient elimination of a complementing plasmid from a knock-out strain which can occur while the strain is being made transformation-competent. Efficient plasmid elimination is an essential prerequisite for the high performance of the system during evaluation of large libraries under stringent conditions because elimination of the wild-type gene from the cells prior to introduction of variants prevents recombination-based background growth. The major shortcoming of conventionally used bleach-out systems is indeed due to recombination with a silenced wild-type gene during selection. Even in a *recA* strain, RecA-independent recombination between homologous regions occurs with frequencies between 10^-3^-10^-4^[27]. As soon as the number of expected functional variants within a library drops below 10^-3^ selection is primarily for recombination events instead of functional library members. This can clearly be seen in our selection experiment using the GroEL bleach-out strain SBΔ*recA* and mock libraries with decreasing ratio of functional variants. Even though our strain was deficient for *recA,* we frequently isolated false positive variants.

Besides being recombination-free, another advantage of the presented set-up over bleach-out systems is that selected variants are directly expressed in a clean strain-background circumventing laborious post-transformation work such as P1-transductions. This allows for subsequent purification of engineered proteins for *in vitro* characterization and user-defined *in vivo* applications without running the risk of wild-type contamination.

We also show that even after elimination of the complementing vector from the knock-out strain, cells remain viable for one or more generations - depending on the gene - and electrocompetent cells prepared after elimination yield up to 10^7^ transformants μg^-1^ DNA.

As in our set-up the essential target genes are expressed from an inducible promoter during competent cell preparation, appropriate bleach-out times can be adjusted for individual gene products by tuning the inducer concentration.

The system was validated with three different essential *E. coli* proteins: Adk, glyceraldehyde-3-phosphate dehydrogenase and the chaperonin GroEL. Transformation efficiencies and the absence of detectable recombination events proved to be independent of the essential target gene.

## Conclusions

In the current work we present a straightforward, transformation-based system which enables the genetic replacement of a wild-type essential gene of interest by a library or variant. It thereby directly results in the isolation of functional variants in a clean strain background with considerably reduced effort.

Furthermore, it substantially facilitates working with and engineering of essential genes and their protein products making it an experimentally easy, fast and scalable task.

Finally, it should be possible to adapt the here introduced replacement strategy to other hosts – like e.g. yeast, *Bacillus subtilis* or *Clustridium* spec. - for which homologous recombination-based knock-out strategies are available [28-30].

## Materials and methods

### Chemical and enzymes

Restriction enzymes and ligase were obtained from New England Biolabs (Ipswich, MA, USA) and used according to manufacturers’ instructions. Chemicals were purchased in the highest purity available from Sigma-Aldrich, Fluka (Buchs, Switzerland) or Roth (Lauterbourg, France). Trypton and yeast extract were from BD Bioscience (Basel, Switzerland). Oligonucleotides and Sanger-sequencing service were purchased from Microsynth (Balgach, Switzerland).

### Strains and plasmids

*E. coli* DH10B was used for general cloning procedures. *E. coli* W3110 was used as the chassis for all chromosomal deletions (see Table 1 for an overview on strains used in this study). SBΔ*recA* is a derivative of strain MGM100 [10]. It was constructed from BW25113 *recA::*FRT [1] by P1-phage transduction using a lysate from MGM100 and selecting for *Km*^*R*^. The final clone was confirmed by PCR analysis of the *groE* and *recA::FRT* locus and by its inability to grow on glucose after GroEL-bleach-out. Plasmid pKOCOMP-*adk* is a derivative of pKD46 [17] and was constructed by first cloning the *adk* gene into the multiple cloning site of expression vector pACT3 [31] via restriction sites *Bam*HI and *Hin*dIII using primers pKOCOMP-*adk*-fw and pKOCOMP-*adk*-rv (see  Table S1 for primer sequences). The resulting vector pACT-*adk* was used as template to amplify the P*tac* promoter-controlled *adk* gene with primers pACT-forward and pACT-reverse, encoding for a I-*Sce*I recognition site, and cloned into pKD46 via its unique *Nco*I-site. For construction of pCOMP-*adk* the P_*tac*_ promoter-controlled *adk* gene was amplified with primers pACT-SceI-Spe and pACT-Pac and the PCR product was cloned into the unique *Spe*I and *Pac*I sites of pSEVA432 (*ori* pBBR1, Spec^R^ resistance)*.* Plasmid pSEVA132-*adk* is a derivative of pSEVA132 (*ori* pBBR1, Ap^R^) and encodes *adk* controlled by its natural promoter and fused to a C-terminal 6xHis-tag. It was amplified from genomic *E. coli* DNA using primers *adk*-forward and *adk*-reverse and cloned into pSEVA132 via restriction sites *Xma*I and *Sac*I. Plasmid pCOMP-*gro*E was constructed by amplification of the P_*tac*_-controlled *groE*-operon from pACT-ESL using primers pACT-SceI-Spe and pACT-Pac and cloned into the unique *SpeI* and *PacI* sites of pSEVA431. pACT-ESL is pACT3 derived and encodes the P_*tac*_ controlled groE operon. *groS* was amplified from W3110 by PCR with primers *groS*-fw and *groS*-rv and *groL* was PCR-amplified with primers *groL*-fw and *groL*-rv and then sequentially cloned into the *Kpn*I and *Hin*dIII sites of pACT3. Plasmid pSEVA132-*groE* was constructed by amplification of the natural *groE* operon from *E. coli* genomic DNA using primers *groE*-forward and *groE*-reverse and cloning them into the unique sites *Xma*I and *Xba*I. Plasmid pCOMP-*secBgpsA* was constructed by cloning the natural *secB-gpsA* transcriptional unit, amplified with primers *secBgpsA*-forward and *gpsA*_I-*Sce*I-reverse, into pSEVA431 via restriction sites *Xma*I and *Xba*I. Plasmids pSEVA132-*adk*^*stop*^, pSEVA132-*adk*^*watermark*^, pSEVA132-*groE*^*stop*^, pSEVA132-*groE*^*watermark*^ and pSEVA132-*secBgps*A^stop^ were constructed by amplification and re-ligation of pSEVA132-*adk*, pSEVA132-*groE* or pSEVA132-*secBgps*A using primers *adk*-stop-fw/*adk*-stop-rv, *adk*-watermark-fw/*adk*-watermark-rv*,* groE-watermark-fw/groE-watermark-rv, groE-stop-fw/groE-stop-rv and secBgpsA-stop-fw/secBgpsA-stop-rv.

Helper plasmids pI-*Sce*I and pP_*ara*_I-*Sce*I are derivatives of pSEVA671 (*ori* p15A, Gm^R^). The gene for I-SceI nuclease was amplified from plasmid pSTKST [32] using primers I-SceI-fw and I-SceI-rv and cloned into pSEVA671 via *Pac*I and *Eco*RI restriction sites. The RhaR-RhaS/P_*rha*BAD_ regulatory system was amplified from *E. coli* genomic DNA using primers Rha-forward and Rha-reverse and cloned in front of I-SceI via restriction sites *Nsi*I and *Spe*I. The AraC/P_*ara*BAD_ regulatory system was amplified from *E. coli* genomic DNA using primers ParaBAD-fw and ParaBAD-rv and used to exchange RhaR-RhaS/P_*rha*_ using sites *Nsi*I and *Spe*I.

### Preparation of competent cells and transformation

*E. coli adk::kan* [pKOCOMP-*adk*, pI-*Sce*I] or [pCOMP-*adk*, pP_*ara*_I-*Sce*I] were grown overnight in LB liquid broth supplemented with 50 μg mL^-1^ kanamycin, 10 μg mL^-1^ gentamicin, 100 μM IPTG and 0.5% (wt/vol) glucose (to efficiently repress I-*Sce*I production) at 30°C or 37°C. Cells were pelleted, washed once with LB and diluted 1:100 in fresh LB broth, supplemented with the same antibiotics as before but without glucose. Cells were grown at 37°C. At an OD_600_ of 0.2, 10 mM rhamnose or 0.2% arabinose (wt/vol) was added to induce I-*Sce*I nuclease production. When reaching an OD_600_ of 0.4-0.5, cells were chilled on ice for 30 min, harvested and washed twice with chilled water and once with 10% glycerol as described [26]. For transformation with the test plasmids and the mock libraries, 50 μL cells (OD_600_ around 100) were mixed with 1.5 ng DNA, exposed to an electrical pulse of 1.3 kV and recovered in 1 mL LB broth supplemented with 10 mM rhamnose or 0.2% arabinose for 1 h at 37°C. Selection was done overnight at 37°C on LB agar plates containing 50 μg mL^-1^ kanamycin, 10 μg mL^-1^ gentamicin, 100 μg mL^-1^ ampicillin and 10 mM rhamnose or 0.2% arabinose. The GroEL-specific selection system based on W3110 *groE::kan* [pCOMP-*groE*, pP_*ara*_I-*Sce*I] and the GpsA-specific selection system based on W3110 *secBgpsA*::kan [pCOMP-*secBgpsA*, pP_*ara*_I-*Sce*I] were treated the same way. For preparation of competent SBΔ*rec*A, cells were grown overnight in LB supplemented with 50 μg mL^-1^ kanamycin and 0.2% arabinose. Cells were washed twice with water and diluted 1:100 in fresh LB medium. When cells reached an OD_600_ of 0.1 0.5% glucose was added to repress chromosomal GroEL production. Cellular GroEL was bleached for two generations before cells were harvested for competent cell preparation (at OD_600_ of 0.4).

### Determination of plasmid loss

Loss of pKOCOMP-*adk* or pCOMP-*adk,* pCOMP-*groE* and pCOMP-*secBgpsA* was determined as follows: The corresponding knock-out strains *adk*::*kan*, *groE*::*kan* and *secBgpsA*::*kan* containing helper plasmid pI-*Sce*I or pP_*ara*_I-*Sce*I were grown in LB liquid broth supplemented with 50 μg mL^-1^ kanamycin and 10 μg mL^-1^ gentamicin until exponential growth was reached. Next, I-*Sce*I nuclease production was induced with 10 mM rhamnose or 0.2% arabinose. After induction aliquots were taken after 0, 10, 20, 40, 60, 120 and 180 min and normalized to OD_600_. Serial dilutions were subsequently plated on LB agar supplemented with 0.5% glucose. The next day the number of colony forming units (CFU) was counted. The number of colonies resulting from aliquots which had been taken from a control culture grown in the absence of rhamnose or arabinose were set to 100% CFU.

### Chromosomal knock-out of essential genes

Knock-outs were done by λ red-based recombination with a PCR-encoded kanamycin resistance cassette as described earlier [17]. The kanamycin-cassettes were generated with pKD13 as a template and primers *adk-*H1 and *adk*-H2, *groE*-H1 and *groE*-H2 or *secBgpsA-*H1 and *secBgpsA*-H2. Knock-outs were PCR-verified with primers P1-P6 as indicated in Additional file2 Figure S1 and Additional file 1 Table S1.

## Competing interests

The authors declare that there are no competing interests.

## Authors’ contributions

SB performed the experiments, SB and SP designed the experimental approach and wrote the manuscript, SP supervised the research. All authors read and approved the final manuscript.

## Supplementary Material

Additional files file 1 Table S1. Primers used in this study. Click here for file

Additional files file 2 Figure S1. PCR verification of knock out strains. Click here for file
