# Supplementary material for: A genetic replacement system for selection-based engineering of essential proteins
Source: Microb Cell Fact. 2012 Aug 16;11:110. doi: 10.1186/1475-2859-11-110 (PMC3503863; doi:10.1186/1475-2859-11-110)
Supplement: Additional files file 1 — Table S1. Primers used in this study. [file 1475-2859-11-110-S1.docx]

**Supplemental Table 1. Primers used in this study**

| **Primers** | **Sequence** |
| --- | --- |
| **Cloning** |  |
| pACT-foward | ATGCCCATGGCAGGCAGCCATCGGAAGCTG |
| pACT-reverse | ATGCCCATGGCTAGCATGAGCGGATACATATTTGAATG |
| pACT-SceI-Spe | CTACTAGTCCTGCAGGAGTTACGCTAGGGATAACAGGGTAATTATAGCCCGTTCTGGATAATGTTTTTTGCGC |
| pACT-Pac | CCTTAATTAAGAGATTACGCGCAGACCAAAACG |
| pKOCOMP-adk-fw | CTGGGATCCAAGAAGGAGATATACATATGCTCGAGCGTATCATTCTGCTTGG |
| pKOCOMP-adk-rv | GTCAAGCTTTTAGTGGTGGTGGTGGTGGTGTCTAGAGCCGAGGATTTTTTCCAGATCAGC |
| adk-forward | GGATCCCGGGCCGCAAATTATCTCGCCATTAACCG |
| adk-reverse | CGGAGCTCTTAATTAACGGCCGGCTTAGTGGTGGTGGTGGTG |
| adk-stop-fw | TAATAATTCGACGTACCGGACGAACTG |
| adk-stop-rv | AACGTAATCAACATTGATGCCCGCTTC |
| adk-watermark-fw | GAAAAACTGTATTTTCAGGGGACCGGTGAAGAACTGACTACC |
| adk-watermark-rv | CGGCGGATTGAATTTAACGTG |
| groE-forward | CCGTCTAGACACCCGATTTTGTGCTGATCAGAAT |
| groE-reverse | GACGGCGATGAAGAAATTGCGATGAAATGTGAGGTGAATCAGGGTTTTCACCTGTGTAGGCTGGAGCTGCTTCG |
| groL-fw | GCTCTCTAGACACGACACTGAACATACGAATTTAAG |
| groL-rv | GATCAAGCTTTTACATCATGCCGCCCATGCC |
| groS-fw | ATGCGGTACCCTTTCTCAAAGGAGAGTTATCAATGAATATTC |
| groS-rv | CGTGTCTAGATTACGCTTCAACAATTGCAGAATG |
| groE-stop-fw | TGAACCGTGATCTCTGAAGAGATCG |
| groE-stop-rv | GCCAGTCAGGGTTGCGATATCC |
| groE-watermark-fw | TGGTCTCACCCGCAGTTCGAGAAGTCGGGCGGCGGCACCGTGATCTCTGAA GAGATCGG |
| groE-watermark-rv | GCCACCGCCGATCACGGTACCGCCAGTCAG |
| secBgpsA-forward | CGCCCGGGCCATGGGTGTGAACGTTGGCATTACATTGCG |
| secBgpsA-reverse | GTCTCTAGATTACTTAGTGGTGGTGGTGGTGGTGGTGGCTGCTGCGCTCGTCC |
| secBgpsA-stop-fw | TAATAAGGCAAAAGTTTCCGCGTTTACAGC |
| secBgpsA-stop-rv | GCAGTGCAGCAGCTGCTGG |
| gpsA_I-*Sce*I-reverse | GTCTCTAGATCTATATTACCCTGTTATCCCTAGCGTAACTTACTTAGTGGCTGCTGCGCTCGTCC |
| I-SceI-fw | CTTTAATTAAACGTCGGGCCCTTATTTCAGGAAAGTTTCGGAGGAGATAG |
| I-SceI-rv | CGGAATTCCTATATGCATATGAAAAACATCAAAAAAAACCAGG |
| Prha-fw | CGTACTCTCTAGACCATGCATATCTATATCTCCTTCTTAAAGTTAAAC AAAATTATTTCCGACCTTCTCGTTACTGACAGGAAAATGGG |
| Prha-rv | CCAGACTAGTCCGGCGCAGTATGACTACATCAGTTGG |
| P*ara*BAD-fw | CAGACTAGTCTCCGTCAAGCCGTCAATTGTC |
| P*ara*BAD-rv | GCATATAGGTACCCTCCTGCTAGCCCAAAAAAACGGG |
|  |  |
| **Knock-out and verification** |  |
| adk-H1 | GGTATCGTTTATCGCTTTTTCAAAAAATTCGACACATTTTAAGGGGATTTTCGCAGTGTAGGCTGGAGCTGCTTCG |
| adk-H2 | CACCCTAACCCTCTCCCCGAGGGGGCGAGGGGACTGTCCGTGCGCGCTTTCGAAATTCCGGGGATCCGTCGACCTG |
| secBgpsA-H1 | GATGACTTGTATGCATTGGATGCACGTGGTGGACTGGATCCCCTGCTGAAATAACATTCCGGGGATCCGTCGACCTGC |
| secBgpsA-H2 | CCGGCCCGTTCTGCGCGGGCCGGGTCATAGCGGTAACAAAGGTTCCCTGGGGTGTAGGCTGGAGCTGCTTCG |
| groE-H1 | GGCGTCACCCATAACAGATACGGACTTTCTCAAAGGAGAGTTATCAATGAATATGTGTAGGCTGGAGCTGCTTCG |
| groE-H2 | CCCGGGGGTTTGTTTATTTCTGCGAGGTGCAGGGCAATTACATCATGCCGATTCCGGGGATCCGTCGAC |
| P1 | CTTCGTGTCATCCGGCATTTTTCTTTTCATCATCTGC |
| P2 | GCGGAAAAGTGAGGAAGAAGAACTAATTGCTG |
| P3 | CGCCCGGGCCATGGGTGTGAACGTTGGCATTACATTGCG |
| P4 | GTCTCTAGATTACTTAGTGGTGGTGGTGGTGGTGGTGGCTGCTGCGCTCGTCC |
| P5 | GGTCACCAGCCGGGAAACC |
| P6 | CGTCCACAATGCGTACACTCTG |
| P7 | GCGAAATGCTGCCGGTTCTGG |
| P8 | TCAGTCGGCAAAATTTCGCCAAATCT |
| P9 | ATGACTGACAGTGAACTGATGCAGTTAAG |
