# Supplementary figures and images for: A genetic replacement system for selection-based engineering of essential proteins
Source: Microb Cell Fact. 2012 Aug 16;11:110. doi: 10.1186/1475-2859-11-110 (PMC3503863; doi:10.1186/1475-2859-11-110)

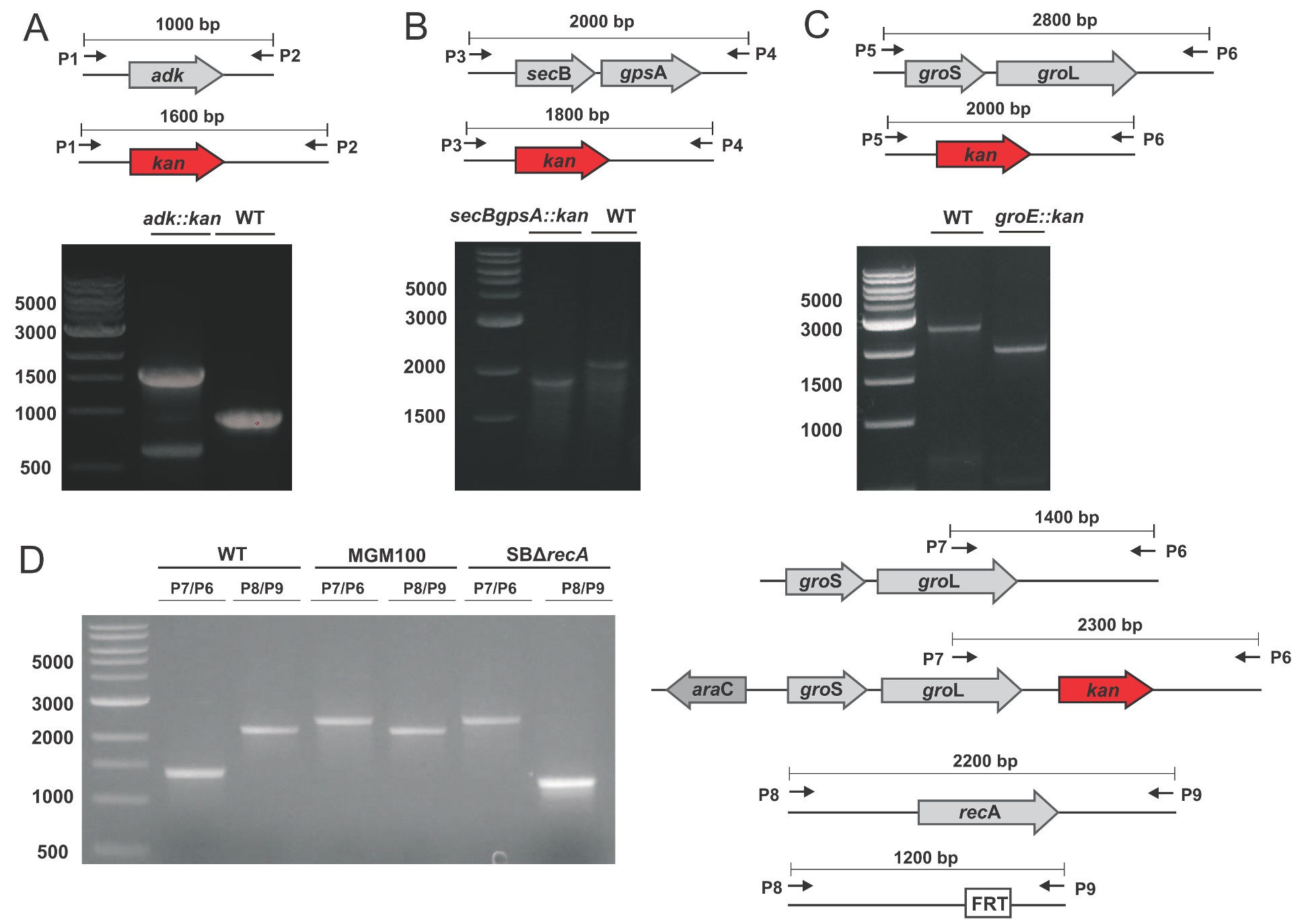

Supplement: Additional files file 2 — Figure S1. PCR verification of knock out strains. [file 1475-2859-11-110-S2.tiff]
